# The University as a Source of Social Capital in Chile

**DOI:** 10.3389/fpsyg.2021.601143

**Published:** 2021-02-05

**Authors:** Pascale Labra, Miguel Vargas, Cristián Céspedes

**Affiliations:** ^1^Facultad de Economía y Negocios, Universidad Andrés Bello, Santiago, Chile; ^2^Head of Foreign Languages Unit, Facultad de Administración y Economía, Universidad de Santiago de Chile, Santiago, Chile

**Keywords:** belongingness, networks, segregation, friendship, homophily

## Abstract

This paper investigates the structure and composition of the social network formed on the campus of the Faculty of Economics and Business of Diego Portales University, Chile, exposing a series of characteristics that are aligned with similar research in the field of networks. We use a model of social networks formation in order to understand socioeconomic and academic factors that predict the formation of friendship between two students. Specifically, we test empirically our model, using students' administrative information. Of special interest is the impact of the length of stay of the students in the university, with which we refer to the years completed in the degree course, in the probability of establishing friendship ties where being socioeconomically different is a condition. The mechanism behind a result like this is the sense of belongingness that being part of the same institution may induce amongst students. By means of counterfactual simulations we found evidence in favor that passing through the university increases the probability of forming friendship networks, which can mean a kind of social capital, thus reducing socioeconomic segregation from the Chilean school system. Given the importance of this finding, we believe that policies that increase the sense of belongingness such as cultural events, leaderships programs, and community should be implemented on university campuses.

## 1. Introduction

The goal of this investigation is to cast light on the effect that university has upon the chance of befriend students from different socioeconomic backgrounds. Chilean cities and schools present high levels of socioeconomic segregation (Lambiri and Vargas, 2011; Elacqua, 2012; Trevino et al., 2014). The latter changes when students arrive into university, a place where is possible to observe more mixed communities (Espinoza and González, 2012). However, given the fact that at this point, from neighborhoods and schools, student have already form their social networks one would ask if it will be enough sharing a university ward with students from different socioeconomic status to modify their social networks.

The answer is not necessarily clear at first sight. On one hand, there is significant evidence regarding the tendency of individuals with similar characteristics to form ties. For instance, it has been found that the social origin is an important driver to induce homophily in university learning networks (Xu and Weinberg, 2014; Weber et al., 2020). In the same way, networks analysis and their different components are marked by the actors tendency to establish relationships in terms of how similar they are (Lazarsfeld and Merton, 1954; Currarini et al., 2009), and for this reason the concept homophily is widely linked to a network's segregation levels (Blau, 1977; McPherson et al., 2001). Homophilia's level on the process of bounding plays a key role, as it determines the way people gather, exchange information, and make decisions, what is not irrelevant when it comes to networks shaped up by race features, income level, religious belief, and educational background (Currarini et al., 2009). High levels of homophily caused distance among actors within a social network, and this distance does impact on integration. On the other hand, university may induce a sense of belongingness, a concept that lies on people's need of bounding, where those bounds will embody the boost individuals need for their self-development, being this the result of the experience and interaction with their environment (Baumeister and Leary, 1995; Den Hartog et al., 2007; Brechwald and Prinstein, 2011). In this line of argument, it has been found that the sense of connection to one's university buffers racism and provides a base for exploration of cross-cultural relationships amongst international and domestic students in American universities (Glass and Westmont, 2014).

On this regard, belongingness means to humans their need of being accepted, being able to identify and be identified, as well to recognize and be recognized by their partners as part of a whole (Maslow, 1970; Baumeister and Leary, 1995; Yuval-Davis, 2006; Brechwald and Prinstein, 2011; Mahar et al., 2013). According to literature, belongingness is a society's main motivation to gather, therefore bounding is essential for belongingness theory, being critical for individuals bounding, communication, and social relationships along their lives (Maslow, 1970; Baumeister and Leary, 1995; Den Hartog et al., 2007). As a result, belongingness is directly linked to individuals interaction inside a social network, that is to say, the way they teamwork and the results individuals achieve, the way they collaborate when part of a group, the status they can achieve as part of society, besides peers effect and influence within educational contexts (Den Hartog et al., 2007; Brechwald and Prinstein, 2011). Within groups with high levels of segregation and homophilia, belongingness is critical for reducing distance among sub groups in a social network, especially for education contexts analysis, where networking relies on individuals influence on their peers (Baumeister and Leary, 1995; Choudhury et al., 2006; Brechwald and Prinstein, 2011). Aspects such as the dissemination of information, investment and risk decisions, access to jobs, commerce, education, opinion formation and social mobility, to name a few, are affected by the degree to which society is segregated (Montgomery, 1991; Granovetter, 1995; Calvó Armengol and Jackson, 2004; Currarini et al., 2009), considering the latter as the dispersal of a particular group in a geographic area or in a certain situation (Royuela and Vargas, 2006). In this sense, a society where there is a greater degree of social mobility, the relevance of the socioeconomic origin of individuals would not represent an obstacle to accessing a set of new and better opportunities.

This is an important subject of research because networks play a central role in a series of relevant aspects of individual and collective action, permeating the social and economic life of individuals. Several studies have shown the relevance that social networks have on individuals well-being (Calvó-Armengol et al., 2009; Lee et al., 2011; Fletcher and Ross, 2012) and on learning, information transmission and labor market outcomes at the beginning of professional careers (Mayer and Puller, 2008). Summing up, the innumerable ways in which network structures influence people's well-being make it essential to understand both their impact on behavior, as well as to identify those patterns of network structures that are likely to be observed in a society (Currarini et al., 2009).

## 2. Literature Review

### 2.1. Social Networks Formation

The Chilean educational system is very segregated as it going to be explained later on, something that is particularly true at school level. The latter changes at some extent in universities (Espinoza and González, 2012). However, the fact that students in university have the chance to interact with more mixed communities does not guarantee that they will intermingle with students of different socioeconomic backgrounds, or in other words that their social networks will significant change once they are in the university. Understanding how social networks formation work in university arises as an important task because networks and their structure is a determining factor in the way a society works and they have an important effect on individuals well-being. Social networks are key elements in societies because they are device which allows the exchange of information, culture, knowledge and social capital and social support (Yuan et al., 2018).

The importance of social networks also falls on the labor markets, this being well documented in a series of studies present in the associated literature. There is considerable evidence regarding the role that social media plays in job search. A vast body of research in economics and sociology has shown and concluded that at least 50% of jobs are found through informal channels, such as friends, family and general social contacts (Montgomery, 1991; Granovetter, 1995; Calvó Armengol and Jackson, 2004). The exploration of the vast majority of these studies with different types of occupations and varied levels of education and income, present similar figures that support the relevance of social contacts in terms of employment (Calvó Armengol and Jackson, 2004). Therefore, the exchange of information is more likely to be more productive, regarding labor supply, when two agents are closer in terms of their respective occupations and ties (Conley and Topa, 2003). Better connected individuals will have the opportunity of having access to new information sooner and at the same time they will be able to have high level of influence on the eventual spread of information (Jackson et al., 2017). There are investigations on the opposite causality as well: unemployment induces social withdrawal particularly for people above 50 years of age (Von Scheve et al., 2017; Rozer et al., 2020).

Consequently, social networks matter and universities are an important place where they can be formed. First, because, something that is particularly true in Chile, probably it will be the first place where a significant amount of students will have the chance of interact, for first time, with people of a different socio-economic background, something that does not guarantee that these interactions will have as a result the formation of friendships. Second, because, it has been documented that labor market connections and business partners networks are formed using knowledge from previous social interactions as those that arise in universities (Ioannides and Loury, 2004). As a matter of fact, at least in U.S.A., universities have made an effort in order to implement different policies to facilitate the integration of students of diverse race, nationalities and socio-economics background (Mayer and Puller, 2008).

The formation of social networks has been studied both theoretically and empirically. On the theoretical side, the traditional approach has been using game theory and agent-based models which propose micro level rules for the formation of connections and the they prove that these rules have implications for the macro level properties and structures (Calvó Armengol and Jackson, 2004; Jackson, 2008; Yuan et al., 2018). On the empirical side, using game theory models as a base, several investigations have analyze the networks formation with focus on the identification of those characteristics that are the drivers of the link formation (Mayer and Puller, 2008; Smirnov and Thurner, 2016; Mele, 2017). Some results that have been found regarding the networks formations in universities indicate that this type of social networks in U.S.A. are strongly determined by factors such as race, as matter of fact, blacks and Asians have disproportionately more same race friends than would arise from the random selection of friends, even after controlling for socioeconomic background, ability, and college activities (Mayer and Puller, 2008). It has been shown as well that academic homophily (this concept is explained in the next subsection) is a result of selection because students prefer to reorganize their social networks according to their academic performance, instead of adapting it to the level of their local group and there is no evidence of a pull effect, i.e., a social environment of good performers that would motivate bad students to improve their academic outcomes (Smirnov and Thurner, 2016).

The present investigations belongs to this second branch. Based on a simple theoretical model, we study, empirically, the forces behind the social networks formation in the Chilean higher education system context, trying to identify the factors that explain the friendship between students such as academic performance, gender, age or socio-economic background. As we do not have experimental data, we conduct a series of counterfactual simulations in order to test our hypotheses.

### 2.2. Segregation, Social Networks and Homophily

The degree to which a society is segregated throughout the network can be critical when determining aspects such as how fast information is disseminated or what the level of underinvestment in human capital will be, among other things (Currarini et al., 2009). This is why in the social media literature the phenomenon of homophilia is recurrent. The term is defined as the tendency of people to establish relationships with those who share similar characteristics (Lazarsfeld and Merton, 1954; Currarini et al., 2009). The level of homophilia that exists in a given network is of great importance in the speed at which a society reaches a global consensus, which becomes more relevant when the agents' decisions are complementary, so the authors argue that studying and understanding homophily is crucial to understanding the functioning of a society (Calvó Armengol and Jackson, 2004; Golub and Jackson, 2012). Homophilia is also closely linked to segregation. High levels of homophily imply high segregation on social media across a variety of basic demographic states such as race, ethnicity, age, education, and income (Blau, 1977; McPherson et al., 2001), thus influencing phenomena such as social isolation, which in turn is theoretically linked in contemporary literature with issues of social inclusion, inequality and poverty (DiPrete, 2011). There is an extensive literature that has investigated the phenomenon of homophily according to characteristics such as age, race, gender, religion, and profession, generally being a strong and robust observation (Currarini et al., 2009). Empirical evidence regarding homophilia and segregation indicate significant levels for both phenomena in both adolescents and adults. An example of this is indicated by Currarini et al. (2009), who conducted a friendship network analysis of a representative sample of high school students from schools in the United States. They used the national Add Health 2 survey as a data set and identified homophily patterns in the network. Specifically, they point out three important observations, the first of which shows that those groups of a greater relative size in the population have a greater tendency to form bonds of friendship with those who are of the same type, considering type according to race. As a second observation, the authors conclude that these same groups of greater relative size tend to form significantly more friends per capita. As a third point, they reveal that those groups of smaller relative size in the population tend to integrate more effectively, with minorities being less racially segregated groups than those that represent a higher percentage of the population. Similar results are found in recent studies on racial and ethnic homophilia, mainly in the United States (Moody, 2001; Mollica et al., 2003; Currarini et al., 2009). High levels of homophily may guide groups to be insular and to act different from others groups, generates poverty traps and underinvestment because of complementarities in behavior, and makes information diffusion slower across groups (Jackson et al., 2017). Associated with the phenomena of homophily and segregation arises the concept of social capital. In the search for the definition of social capital, we find different perspectives and a series of concepts that basically refer to the resources that the actors of society can mobilize as a consequence of their belonging to a group. This extends to the fact that in these relationships there is a certain degree of trust, solidarity and reciprocity, therefore, culture and institutions are a relevant factor in this dynamic, in addition to which their training would be linked to individual characteristics, thus as well as contextual variables such as inequality, racial diversity, institutions and political designs (Boix and Posner, 1996; Adler and Kwon, 2002; Durlauf, 2002). One of the first definitions of social capital indicates that they are those characteristics of a social organization, such as networks, norms and trust, that facilitate coordination and cooperation among its members in order to achieve a common benefit (Putman, 1995), reason why social capital as the connection between individuals in their social networks, and understood as a rich resource, is a factor that would contribute to the development of the well-being of agents in a more virtuous way than in those societies where individuals are segregated. Later, following the same line, the literature indicates the importance of the use and accumulation of social capital as a resource that facilitates the lives of individuals and allows them to reconcile individual interest with common interest (Putman, 1995). Social capital can also be defined as a set of values or norms shared among members of the same group that allows them to cooperate with each other informally or circumstantially. Trust in this interaction would be essential in the path toward more efficient organizational functioning (Fukuyama, 1997). (Lazarsfeld and Merton, 1954) classifies homophilia according to two parameters, value and status. Homophily of value is related to the personality, attitudes, aspirations and future expectations, while status homophilia is based on intrinsic characteristics of individuals such as age, ethnicity, religion and gender, or acquired characteristics such as education, profession or occupation. An interesting bridge can be established between the research question posed and the importance of social capital. The interaction between individuals of different socioeconomic levels and the information they share, given the bonds of friendship that are formed through the network in the university, can mean a relevant source of social capital, especially for those who in the first instance were more segregated, this prior to entering the institution.

### 2.3. Segregation in Chilean Educational Institutions

Chilean cities are segregated (Lambiri and Vargas, 2011). This spatial inequality is represented in the educational system as well. There are several reasons behind this fact. First, as mentioned, cities and neighborhood in Chile are segregated, hence schools in well-off neighborhoods have a greater share of students coming from high income households, meanwhile schools in poor neighborhoods the composition is the opposite. For instance, some investigations have shown a comparison between different countries of the school segregation by aggregate socioeconomic status (SES), the result is that school segregation level is much higher in Chile than the one observed in countries such as USA, Brazil, and Argentina (Chmielewski and Savage, 2014; Allende et al., 2018). Second, the framework of the Chilean educational system is quite segmented as well as it is made out of three different type of schools, namely, private, subsidized, and public. The first group corresponds to fully private school where tuition fees are fully paid by families. In the second groups, the Government gives a voucher to households so they can choose from a group of private schools, not any school but subsidized schools, and the third group correspond to schools fully financed by the Government, as a matter of fact these type of schools since the reform in the 80's have depend directly from municipalities. Therefore, on average, high income students go to private school, middle income to subsidized schools and poor students to public schools. There is an important body of research that has described this system and that has studied its consequences on different aspects of students well-being (Saporito, 2003; Schneider et al., 2006; Elacqua et al., 2011; Allende et al., 2018). However, this is not the end of the story. Within the subsidized schools there is another layer of segmentation as well. Families, if they want, can pay an extra amount additionally to the voucher in order to choose a school that charge a greater amount of money than the voucher provides: the co-payment. Consequently, according to the amount of the co-payment, it will possible to observe an additional level of segregation, because within the middle class segment, households will separate each other in different subsidized schools according to the extra money they pay (Mizala and Torche, 2012).

This is particularly important if we consider that the school system represents the first formal approach of individuals to an environment of socialization outside the family^1^. In Chile, this first instance is conditioned to the high level of socioeconomic segregation of the students in the system, mainly when we refer to the most vulnerable and those with better socioeconomic conditions. According to the evidence already mentioned, the educational results of Chilean students are closely linked to the socioeconomic level of the families and the degree of stratification of the school system, given that they tend to attend establishments of similar socioeconomic level, sharing with peers who have similar social conditions and cultures (García Huidobro and Bellei, 2003; Valenzuela et al., 2008). Hence, the Chilean school system, for the moment, does not represent a space in which students from different socioeconomic levels are integrated.

That said, the question arises about the means and instances in which socioeconomic origin is not an impediment to establish relationships that allow agents to form a network of contacts through which access to greater and better opportunities contributes to increase your well-being. It is of particular interest in this research to determine if the university, as a heterogeneous medium, represents a channel through which individuals from different socioeconomic levels manage to interact and establish bonds of friendship, ties that build a network of contacts that means an increase in social capital for the students. The latter is understood as the set of characteristics of a social organization, such as networks, values or norms that are shared among the members of a group and that allow them to cooperate with each other informally or circumstantially. Trust in this interaction would be essential on the way to more efficient organizational functioning (Putman, 1995; Fukuyama, 1997).

The broad theoretical framework on social capital allows us to answer a series of political, social and economic questions, so moving forward in its study represents opening up new possibilities to answers that have not yet been resolved, especially when the objective is to expand social capital in countries where socioeconomic segregation does not allow dynamic social mobility. This research seeks to highlight the friendship networks formed by university students, to later relate them to the socioeconomic level that each one has. This is a first approximation to the diagnosis of the distribution of social capital prior to the world of work.

The sample corresponds to Commercial Engineering (BA in Economics) students from Diego Portales University who self-report their five best friends within the School of Economics and Business, this through a friendship survey applied to students from first to fifth year. Given the segregation from the school system, it is interesting to measure how the proportion of friends who are from a different socioeconomic level changes, for each individual in the sample, throughout the 5 years they remain in the university. The reason for choosing this university is because it presents a blended community regarding social backgrounds of its students.

## 3. Materials and Methods

### 3.1. Data

The central axis of this research is found in the friendship networks formed at the university. In order to identify this network, it is essential to determine the links that the students have formed throughout the years of the program. Given that in Chile there is no network data at the university level, it is necessary to apply a survey to students that allows building the network. Specifically, the questionnaire contemplates that the students report who are their five best friends belonging to the same career, five at most, being explicit that in said nomination the order of proximity is relevant, those who are closest occupy the first places in the list of who answers the questionnaire. As a test of effectiveness of the questionnaire, a test pilot was carried out in June 2017. The questionnaire was applied in person to a sample of 78 students belonging to the second and fourth years mainly. The results indicate that 74.36% reported the maximum of five friends, while regarding the way of responding 5.13% did not manage to do it correctly. During the pilot phase difficulties were established in the transcription and processing of the data, this given the calligraphy of the names and surnames reported. Along with this, it was possible to identify the influence among the students when writing their answers, given the spatial proximity when responding. Both difficulties are expected to generate the least possible bias since the final questionnaire will be sent personally to each student via email. In October 2017, the final questionnaire was sent to 1,500 Commercial Engineering students from the Diego Portales University. The data collection process lasted for a period of 3 weeks, a period in which the survey was made available to students through a link sent to the personal email registered by the university. The identification and liaison between those who answered the questionnaire with those who were reported as friends is of fundamental importance and became one of the first challenges of the investigation. The first objective was to identify those who answered the survey, for this, we have a database that contains administrative information about the students. The crossing of said base with the list of registered emails allowed us to identify it. The second objective was to identify the reported students as friends. This stage required more work since the students only reported the first and last names of their friends, often misspelled or repeated, with the latter we mean students with the same name and surname. This process requires the verification of the names one by one, comparing both databases. Finally, a network of 965 nodes and 1,510 links was built. In addition to the aforementioned information, a set of data with academic and socioeconomic characteristics of the students is available. Said information was granted by the university to be used only for research purposes, a rule that is stipulated by contract. The variables used are gender, year of admission to the university, values correspond to the period 2013 to 2017, academic ranking, type of school from which each student comes, that is, municipal, subsidized or private, decile, type of financing of the program and commune of residence.

As a second source of information, we have an administrative database that contains the students' socioeconomic characteristics and academic results. Used variables include gender, year of admission to the university, values correspond to the period 2013 to 2017, academic ranking, type of school from which each student comes, that is, municipal, subsidized or private, decile (referring to the students' family income), type of financing of the program and commune of residence. One of the variables used to build the students' socioeconomic profile is the type of school. We talk about municipal school to refer to those with full public financing. These establishments are mainly assisted by students coming from families places in the low and medium of the country's income distribution. State-subsidized schools are those with mixed financing, both public and private, where students from medium income families assist. Particular or private schools, are fully privately funded, and composed of students from families whose income are in high levels of the distribution system. Within the Chilean school system, 36% of students goes to municipal establishments, while 54% goes to private or state-subsidized schools. Table 1 presents a description of these variables^2^.

**Table 1:** Variables description.

Year of admission	Students' college entrance year.
*Type of school*	Kind of financing of the student's school.
Municipal	Public schools.
Subsidized private	Schools financed by The State and privates, that is to say, mix-financing modality.
Private	Private schools, that is to say, those ones financed by the students‘ families.
*Type of financing*	Type of financing of the program
Private	Category where student's college is paid by their families or by themselves.
Private and CAE	Category where students finance their studies through CAE plus student's own resources or their families'.
Scholarship and CAE	Category where students finance their college through CAE plus complementary scholarships.
Free of charge	Category where students are benefited with full scholarships.
Decile	Income range of students' families, according to the country's income distribution.
Sector of residence	Areas where students live.
Time at the university	Time students have spent at college.

University access segregation in the country is concerning. Only 34% of students who graduate from municipal schools and participated in the admission test for 2018 was granted entrance at the university where they postulated, vs. 81% of students graduated from private schools that were admitted. Data shows that student distribution in the country's universities shows 23% are from municipal schools, 54% from state-subsidized, 20% from private schools^3^.

In this regard, 16% of our sample is composed of students coming from municipal schools, while 41% is from state-subsidized schools and 40% are students from private facilities. The financing variable is composed of private financing, category in which students finance their university tuition through family income of that of their own. State guaranteed loans, or CAE, is a mixed system where students finance their tuition with a state-backed loan and private co-payment. State-backed loan and complementary scholarship, means that the student funds the tuition with CAE and the copayment is financed by the state scholarship system. Lastly, the gratuity segment includes students whose tuition was fully financed by the state. This last financing system started implementation in 2016.

Within our network, 37% of students finances their studies through private sector funds, 25% uses the mixed system of CAE and private copayment, 18% uses CAE and a complementary scholarship, and 20% has a gratuity benefit. State-guaranteed loans, CAE, are granted to students coming from 80% of families with the lowest income, meaning the first eight distribution deciles. Meanwhile, complementary scholarships are granted to students belonging to 70% of the most vulnerable, meaning the first seven deciles, while gratuity is only granted to the most vulnerable 50%, meaning the first five deciles^2^. The results are presented in Table 2.

**Table 2:** Composition and characteristics of the network.

	**2013**	**2014**	**2015**	**2016**	**2017**	**Faculty level**
**Composition**						
Number of students	136	175	171	247	236	965
Fraction of women	0.40	0.44	0.44	0.41	0.32	0.40
Fraction of municipal school students	0.19	0.13	0.12	0.18	0.19	0.16
Fraction of subsidized private school students	0.32	0.27	0.37	0.41	0.47	0.41
Fraction of private school students	0.48	0.41	0.50	0.37	0.32	0.40
Fraction of students with private financing	0.44	0.38	0.39	0.37	0.29	0.37
Fraction of students with CAE funding	0.21	0.33	0.23	0.21	0.26	0.25
Fraction of students with CAE funding and scholarship	0.27	0.29	0.38	0.05	0.04	0.18
Fraction of students with free funding	-	-	-	0.36	0.42	0.20
**Characteristics**						
Average number of friends	2.82	2.78	2.71	4.09	2.90	3.13
Variance number of friends	4.46	3.16	3.57	3.87	2.61	3.77
Skewness number of friends	1.13	0.55	0.96	0.34	0.56	0.68
Cluster coefficient	0.19	0.23	0.20	0.39	0.29	0.27
Degree correlation	0.22	0.15	0.18	0.03	0.05	0.12

#### 3.1.1. Network Description

The social science literature that has dedicated their research to the study of networks provides a wide variety of tools for characterizing networks, therefore it is necessary to introduce the notation used in this document since we will apply some of these measures (Jackson, 2008; Mayer and Puller, 2008). We consider a field with *n* students, or following the terminology of network analysis, a network with *n* nodes. If students *i* and *j* are friends, then there is a link or connection between them. This relationship is symmetrical, that is, if student *i* reports being a friend of student *j*, then student *j* is also a friend of student *i*, this characteristic is characteristic of non-directed networks. The friendships between the students are contained in a matrix g of dimension *n*×*n*. If students *i* and *j* are friends, the components *g*(*i, j*) and *g*(*j, i*) of the matrix are equal to one, otherwise the elements of *g* are zero (Jackson, 2008). Research on networks indicates that these are characterized by a series of patterns in common, among them we can mention the width of the queues in the distribution of the number of friends whose bias tends to be to the right, the cluster coefficients cannot be explained by the random formation of the links and the correlation between the number of friends of the individuals, degree correlation, is positive, this means that nodes with many (few) links tend to be connected with other nodes with many (few) links (Newman, 2003; Goyal et al., 2006; Jackson, 2008). One of the measures that we will use in the analysis and characterization of the network is the aforementioned cluster coefficient. This captures the proportion of an individual's friends who are friends with each other (Newman, 2003; Jackson, 2008; Mayer and Puller, 2008), specifically we will use the average of the cluster coefficients calculated for each individual, so for the individual i we define (Jackson, 2008):

(1) $$C(i)=\frac{\sum_{j\neq i,k\neq j,i}g_{ij}g_{jk}g_{ik}}{\sum_{j\neq i,k\neq j,i}g_{ij}g_{ik}}$$

Regarding the characteristics of the network under analysis, the average number of friends varies from 2.71 to 4.09, while the variance fluctuates between 2.61 and 4.46. Furthermore, it is possible to identify a bias to the right of 0.34 to 1.13, this indicates that in the distribution many students have on average few friends, while fewer and fewer students have more friends. Regarding the cluster coefficient, the maximum value is 0.39 while the minimum is 0.19, the literature indicates that larger networks tend to have small cluster coefficients, so this is directly related to the density of the network, that is, networks with higher density have high cluster coefficients (Mayer and Puller, 2008). In our case, the results match with the evidence since the density of our network is 0.008. The degree correlation is positive in all cohorts.

#### 3.1.2. Segregation of the Network

Regarding the analysis of the level of network segregation, the method proposed by Mayer and Puller (2008) is used, who propose a simple and easy-to-interpret measure when comparing the probability that two individuals in a subgroup are friends, with the probability that two random individuals are friends. This measure of relative segregation is independent of the size of the two groups. The relative probability of friendship of two students from a private school, for example, is defined as:

(2) $$\begin{array}{l} Relative\;probability\;of\;friendship\;(private\;\&\;private)\\ \;=\frac{\frac{No\;pairs\;of\;friends\;from\;private\;schools}{Total\;pairs\;private\;schools\;students}}{\frac{No\;of\;pairs\;of\;friends\;from\;any\;type\;of\;school}{Total\;pairs\;students\;of\;any\;type\;of\;schools}} \end{array}$$

Using the variables type of school of origin and university financing system as an approximation to a socioeconomic profile of the students, we document in the upper part of Table 3 that students who come from the same type of school are more likely to form friendship in comparison for those students who come from different types of school, especially these values are higher for the combinations municipal/municipal school and private/private school. Two municipal college students are 1.23 to 1.37 times more likely to form friendships than two random students form friendships, the latter value should be one when the match is random. Similarly, two private school students are 0.95 to 1.51 times more likely to befriend those of the same type. The rest of the categories, cross combinations, have values less than one. Taking a look at the faculty level, the category with the lowest value is municipal/private, 0.58, while the other categories exhibit values close to one. Regarding the segmentation by type of financing, the free of charge/free of charge combination is the most segregated since it is 2.38 times more likely to form friendship, this at the school level. It is important to mention that the social environment of each individual is determined by the probability of forming friendship with an individual of a particular category together with the composition of said category in the population, which is why the fraction of friends from private school in a private school student depends on his relative probability of forming friendship and on the proportion of students from private school in the total population. Thus, the fraction of private school friends of a private school student corresponds to Mayer and Puller (2008):

(3) $$\begin{array}{l} Relative\;probability\;offriendship\\ \;\times \;Proportion\;of\;private\;school\;students\;in\;the\;population \end{array}$$

If the friendships were formed at random, the distribution of characteristics among the friends of any subset of students should be equal to the distribution of these characteristics in the population (Jackson, 2008; Mayer and Puller, 2008; Currarini et al., 2009), this is what the lower part of Table 3 documents. Specifically, 50% of the students belonging to the 2015 cohort come from private schools, however, 76% of their friends at the university also come from private schools. At university school level the results are similar, private school students represent 40% of the student population and the fraction of friends who studied at the same type of school is 50%. For the category of students from private subsidized schools, it is recorded that they represent 41% of the student body with 49% of friends from private subsidized schools. The students of municipal schools, at the faculty level, represent 16% of the population and the fraction of friends who are also from municipal school is 20%. The results coincide with the evidence from similar research in the area of friendship networks, which indicates that those groups of greater size in the population tend to segregate more than those groups that represent minorities, the latter being the ones that integrate better with groups of different characteristics (Mayer and Puller, 2008; Currarini et al., 2009). In the analysis of the variable related to the type of financing that students use to pay for their degree, we can document that the segregation between students with different types of financing is repeated as with the school variable. In particular, for the category of students benefiting from gratuitousness almost double its proportion in the student population corresponds to the fraction of friends under this modality, in the 2016 cohort, the benefited students represent 36%, while the fraction of friends who finance their studies also via gratuitousness is 69%. The same is true for the 2017 cohort, which registers values of 42% versus 88%. The situation is repeated in most cohorts and categories, with the fraction of friends of the same type being greater than the proportion of these in the faculty in most cases.

**Table 3:** Segregation by school of origin and university financing system.

	**2013**	**2014**	**2015**	**2016**	**2017**	**Faculty level**
**Segregation by type of school**						
**Pair of:**	Relative probability of friendship					
Municipal/Municipal	1.39	0.00	1.23	1.37	1.24	1.23
Municipal/Private	1.09	0.79	0.77	0.87	0.74	0.58
Municipal/Subsidized private	0.80	0.98	1.04	1.08	1.25	1.09
Subsidized private / Subsidized private	0.79	1.26	0.97	1.18	1.50	1.21
Subsidized private /Private	0.86	0.77	0.87	0.87	0.77	0.81
Private/Private	1.47	1.51	1.51	1.19	0.95	1.25
**Segregation by type of financing**						
**Par de:**	Relative probability of friendship					
Private/Private	1.62	1.29	1.30	1.00	0.82	1.12
Private/Private and CAE	0.91	1.09	0.98	0.67	0.99	0.88
Private/Scholarship and CAE	1.23	1.08	1.26	0.53	0.34	0.87
Scholarship and CAE / Private and CAE	1.08	1.57	1.62	0.37	0.42	1.02
Private and CAE / Private and CAE	1.28	1.23	0.72	1.30	1.21	1.15
Scholarship and CAE / Scholarship and CAE	1.29	1.74	1.61	0.63	0.00	1.27
Free of charge/Private	-	-	-	1.20	0.99	0.94
Free of charge / Private and CAE	-	-	-	1.26	1.31	1.05
Free of charge / Scholarship and CAE	-	-	-	0.58	0.53	0.36
Free of charge /Free of charge	-	-	-	1.93	2.10	2.38
**Segregation by type of school**						
Fraction of municipal school students	0.19	0.13	0.12	0.18	0.19	0.16
Fraction of municipal schools students friends of students of municipal schools	0.26	0.00	0.15	0.25	0.24	0.20
Fraction of students of private schools	0.48	0.41	0.50	0.37	0.32	0.40
Fraction of private schools students friends of students of private schools	0.71	0.62	0.76	0.44	0.31	0.50
Fraction of subsidized private schools students	0.32	0.27	0.37	0.41	0.47	0.41
Fraction of subsidized private schools students friends of students of subsidized private schools	0.25	0.34	0.36	0.48	0.71	0.49
**Segregation by type of financing**						
Fraction of students with private financing	0.44	0.38	0.39	0.37	0.29	0.37
Fraction of students with private financing friends of students with private financing	0.71	0.49	0.51	0.37	0.24	0.41
Fraction of students financed using Scholarship and CAE	0.27	0.29	0.38	0.05	0.04	0.18
Fraction of students financed using Scholarship and CAE friends of students financed using Scholarship and CAE	0.35	0.50	0.61	0.03	0.00	0.23
Fraction of students free of charge	-	-	-	0.36	0.42	0.20
Fraction of students free of charge friends of students free of charge	-	-	-	0.69	0.88	0.48

*Values in the inferior part of this table were calculated using those categories with the highest index of segregation*.

### 3.2. Methodology and Results

#### 3.2.1. A Model of Social Network Formation

The model used here to explain social network formation is based on what has been done previously in literature (Jackson and Rogers, 2007; Mayer and Puller, 2008). At the beginning it is assumed an unconnected network. Friendship between students *i* and *j* arises after two events: first, two students meet each other with a probability *p*_*ij*_(*Z*_*i*_*Z*_*j*_), where *Z* represents students observable characteristics of their institutional environment such as being part of the same cohort or how many years the students has been in the university, and second, after meeting they choose if they become friends or not. This decision depends on students features which may be both: observable or unobservable. The former is represented by the vector *X* and the latter by the vector *u*. If student *i* become friend of student *j* then she will derive a utility *U*_*ij*_(*X*_*i*_, *X*_*j*_, *u*_*i*_, *u*_*j*_,; β) where vector β represents tastes for the observed characteristics. Let us consider now a cost related to friendship formation *c*, for instance the time needed to become friends. Taking all these aspects into account we will assume that two students become friends if they considers that the utility derived is greater or equal than the cost of a friendship. So we have for any *i, j* that meet:

(4) $$\begin{array}{l} g\left(i,j\right)=I\left(U_{ij}\left(.\right)\geq c_{i}\right)\times I\left(U_{ij}\left(.\right)\geq c_{j}\right)\equiv I\left(f\left(X_{i},X_{j},u_{ij};\beta \right)>0\right) \end{array}$$

*I*(.) is an indicator function. The reduce form function *f* corresponds to the joint choice to be friends. The functional form used in the analysis is given by:

(5) $$\begin{array}{l} Friendship_{ij}=f\left(X_{i},X_{j},u_{ij};\beta \right)\forall i6=j \end{array}$$

Where *Friendship*_*ij*_ is a dichotomous variable that takes value 1 when both individuals are friends and 0 otherwise. Assuming that *u* follows an extreme value distribution, we can estimate the probability of being friend using a Probit model. We consider this analysis as a good predictor of the factors that determine the formation of friendship in the university, however, we must emphasize that we do not suppose such evidence as causal. The selection of students characteristics that are contained in vector *X* is based on literature previous findings (Jackson, 2005; Marmaros and Sacerdote, 2006; Mayer and Puller, 2008; Flashman, 2011). These characteristic may be either intrinsic to each student or institutional. Consequently, vector *X* contains the following characteristics: gender, cohort, academic performance, income decile, neighborhood of residence, type of school, type of financing, and college time.

As our main hypothesis, we test whether studying in the same university increases the probability that two students of different socioeconomic background become friends. We expect that senior students will have a greater probability of befriend students of a different socio economic background. Albeit in this article we do not test any kind of mechanisms, one plausible explanation behind our hypothesis is that being parto of the same university will provide a sense of belongingness that in turn will encourage friendship between students.

In the following section the marginal effect of these variables on the probability of being friend are estimated using a Probit model and after that using counterfactual simulations we test our main hypothesis.

## 4. Results

### 4.1. Empirical Analysis of Friendship Probability

The focus of this research is on the friendship network formed at the School of Economics and Business of Diego Portales University, which has administrative information that provides data on the students referred to the year of admission, gender, ranking of grades in the university, as well as socioeconomic data such as the type of financing employed to pay for the program, type of school from which they come and the commune in which they reside, the latter provide relevant information for the analysis of the central axis of the research that aims to socioeconomic segregation at the university. The sample used contains 965 students who make up the friendship network formed at the school, for whom there is a complete set of information on the variables mentioned above. To quantify the relationship of individual characteristics with friendship formation, all pairs of students were considered, that is, *N*(*N*−1)/2 possible pairs of friendship. The results obtained are detailed in Table 4. When not conditioned by the characteristics of the individuals, the predicted probability that two students form a friendship bond is 0.32%. The first model analyze some of the variables that predict the probability that student *i* and student *j* are friends. Belonging to different years of entering the university, being of a different gender and different ranking are factors that decrease the predicted probability of forming a friendship link. In the following models, the predicted probability of forming friendship when the students are socioeconomically different is evaluated. For this, variables are added that contain information referring to the type of financing for the degree, type of school, decile and sector of residence. All the mentioned variables decrease the probability that individual i and individual j are friends. Having different types of financing decreases the predicted probability to a greater extent than living in different sectors of the city, which in turn is greater than belonging to different deciles. In addition, dummy variables that indicate the existing cohorts were included, this in order to evaluate the behavior of the predicted probability between old students and new students. When evaluating the marginal effect, the coefficients indicate that the probability of forming friendship ties decreases by the same amount. Table 4.1 includes variables that allow a more specific analysis regarding the type of school and type of financing categories. Regarding the type of school from which individuals come, the results indicate that those who come from schools of the same type, that is, the categories both of municipal schools, both of private subsidized schools and both of private schools have a greater probability of being friends, being the last category the one that has a greater effect. In contrast, those particular combinations—municipal and private—subsidized private decrease the predicted probability of forming a bond between both students^4^. Regarding the type of financing of the program, those students who use different financing methods have a lower propensity to form friendship compared to those who use the same method. We must bear in mind that in each of the estimated models the *R*^2^ obtained is low, this should not be surprising since there are several factors that determine the formation of friendship that are not being considered, such as preferences.

**Table 4:** Factors that predict the probability that two students are friends.

Dependent variable = 1 if individual *i* and individual *j* are friends						
Dependent variable mean: 0.0032						
	**(1)**	**(2)**	**(3)**	**(4)**	**(5)**	**(6)**
Constant	−1.978^***^	−1.918^***^	−1.794^***^	−1.734^***^	−1.710^***^	−1.770^***^
	[0.018]	[0.023]	[0.027]	[0.029]	[0.031]	[0.034]
Different gender	−0.303^***^	−0.303^***^	−0.304^***^	−0.305^***^	−0.305^***^	−0.305^***^
	[0.021]	[0.021]	[0.021]	[0.021]	[0.021]	[0.021]
Different income	−1.252^***^	−1.252^***^	−1.259^***^	−1.259^***^	−1.257^***^	−1.278^***^
	[0.028]	[0.028]	[0.028]	[0.028]	[0.028]	[0.029]
Different ranking	−0.129^***^	−0.128^***^	0.130^***^	−0.129^***^	−0.129^***^	−0.123^***^
	[0.020]	[0.020]	[0.020]	[0.020]	[0.020]	[0.021]
Different decile		−0.088^***^	−0.005	−0.004	−0.003	−0.008
		[0.021]	[0.027]	[0.024]	[0.024]	[0.024]
Different sector of residence			−0.221^***^	−0.211^***^	−0.209^***^	−0.199^***^
			[0.027]	[0.027]	[0.027]	[0.027]
Different type of school				−0.115^***^	−0.109^***^	−0.110^***^
				[0.020]	[0.020]	[0.020]
Different type of financing					−0.044^**^	−0.038^*^
					[0.021]	[0.021]
College time						0.007^***^
						[0.001]
R2	0.193	0.194	0.198	0.199	0.199	0.202

*** *Significant at 1%*,

** Significant at 5%,

* *Significant at 10%*.

**Table 4.1:** Factors that predict the probability that two students are friends.

Dependent variable = 1 if individual *i* and individual *j* are friends			
Dependent variable mean: 0.0032			
	**(1)**	**(2)**	**(3)**
Constant	-1.836^***^	-1.574^***^	−1.639^***^
Different gender	−0.308^***^	−0.122^***^	−0.132^***^
Different income	−1.397^***^	−1.397^***^	−1.469^***^
Different ranking	−0.123^***^	−0.122^***^	−0.132^***^
Different decile		−0.006	−0.005
Different sector of residence		−0.205^***^	−0.196^***^
Different type of school		−0.110^***^	-
Private school - municipal friendship			−0.068^*^
Private school - private subsidized friendship			−0.057
Private school - private school friendship			0.050
Municipal school - municipal school friendship			0.031
Municipal school - private subsidized friendship			0.022
Subsidized private school - subsidized private school friendship			0.058
Different type of financing		−0.042^**^	-
Friendship without financing - CAE			−0.102^**^
Friendship without financing - CAE and scholarship			−0.058
Friendship without financing - free of charge			−0.031
Friendship CAE - free			−0.033
Free friendship - CAE and scholarship			−0.023
Friendship both without financing			0.005
Friendship both with CAE			0.024
Friendship both with CAE and scholarship			0.054
Friendship both free of charge			0.009
R2	0.195	0.201	0.210

*** Significant at 1%,

** Significant at 5%,

* *Significant at 10%*.

The following exercise carried out uses model (6), presented in Table 4, and estimates the predicted probability of forming a conditional friendship bond that individuals belong to the 2013 cohort, senior students, and to the 2016 cohort, corresponding to new students in the career. The results are presented in Table 4.2. Again, differences in socioeconomic factors decrease the probability that individual i is a friend of individual j. The marginal effect of time is less when we evaluate the 2017 cohort, while this increases when evaluating the 2014 cohort. That is, new students have a smaller marginal effect on the probability of forming friendship compared to old students, so the permanence at university, measured in career years, favors the formation of links between students.

**Table 4.2:** Factors that predict the probability that two students are friends, cohort 2013 and 2016.

Dependent variable = 1 if individual i and individual j are friends				
Dependent variable mean: 0.0032				
	**Cohort 2013**		**Cohort 2016**	
	**(6)**	**dy/dx**	**(6)**	**dy/dx**
Constant	−1.550^***^		−1.971^***^	
Different gender	−0.329^***^	-0.00045	−0.299^***^	-0.00220
Different ranking	−0.115^*^	-0.00020	−0.135^**^	-0.00139
Different decile	−0.091	-0.00002	0.030	-0.00029
Different sector of residence	−0.157^*^	-0.00011	−0.193^**^	-0.00224
Different type of school	−0.071^*^	-0.00022	−0.084^**^	-0.00083
Different type of financing	−0.079	-0.00001	−0.099^**^	-0.00101
Time at the university	0.199^***^	0.00003	−0.048^***^	-0.00006
R2	0.212		0.235	

*** Significant at 1%,

** Significant at 5%,

* *Significant at 10%*.

In Table 4.3 is presented model (4). In this case dummy variables by students years of entrance are included. The parameters estimated are all positive and significant. Models (5) and (6) additionally consider interaction between time at university and type of school and time at university and type of financing. Results show a negative impact of these two interaction on the probability of being friends, hence despite the time that students have spent at the university, if they have different socioeconomic background (different type of school and different type of financing) the probability of being friend will be reduced.

**Table 4.3:** Factors predicting the probability of two students being friends.

Dependent variable = 1 if individual *i* and individual *j* are friends						
Dependent variable mean: 0.0032						
	**(1)**	**(2)**	**(3)**	**(4)**	**(5)**	**(6)**
Constant	−1.978^***^	−1.710^***^	−1.770^***^	−2.159^***^	−2.170^***^	−2.223^***^
Different gender	−0.303^***^	−0.305^***^	−0.305^***^	−0.308^***^	−0.307^***^	−0.308^***^
Different income	−1.252^***^	−1.259^***^	−1.278^***^	−1.036^***^	−1.037^***^	−1.035^***^
Different ranking	−0.129^***^	−0.129^***^	−0.123^***^	−0.121^***^	−0.121^***^	−0.121^***^
Different decile		−0.003	−0.008	−0.009	−0.009	−0.010
Different sector of residence		−0.209^***^	−0.199^***^	−1.199^***^	−0.199^***^	−0.200^***^
Different type of school		−0.109^***^	-0.110^***^	−0.112^***^	−0.106^***^	−0.019
Different type of financing		−0.044^**^	−0.038^*^	−0.035^*^	−0.026	−0.025
Time at the university			0.007^***^	0.023^***^	0.025^***^	0.025^***^
Dummy by entrance Year						
2014				0.061^*^	0.061	0.166^*^
2015				0.215^**^	0.215^*^	0.241^*^
2016				0.381^**^	0.382^**^	0.448^***^
2017				0.331^**^	0.331^**^	0.376^**^
Time at the university x Different type of school					−0.001^*^	−0.002^*^
Time at the university x Different type of financing					−0.002^*^	−0.001^*^
Entrance 2017 x Different type of school						−0.183^*^
Entrance 2016 x Different type of school						−0.074
Entrance 2015 x Different type of school						−0.038
Entrance 2014 x Different type of school						−0.110
R2	0.193	0.199	0.202	0.204	0.204	0.205

*** Significant at 1%,

** Significant at 5%,

* *Significant at 10%*.

### 4.2. Counterfactual Simulations

Without detracting from the valuable information that we have, it represents a first approach to the formation of networks in the country's universities, and therefore, of utmost importance in future research that will serve as the basis for the formulation and implementation of policies that aim to reduce segregation in the system, we are aware that the nature of our data limits the econometric options that we can resort to answer the question posed. This is why, after the analysis carried out in the previous section, we have decided to follow the method proposed by King et al. (2000)^5^. This proposes statistical simulation as a way to compute amounts of interest considering uncertainty as a factor present, being, therefore, a tool that helps researchers understand statistical models taking full advantage of the information reported by the estimated parameters. The literature indicates that the definition of simulation moves, mainly, between two fundamentals, the first one refers to the manipulation of variables in order to compute amounts of interest and their variations since they have been assigned different values (Kass et al., 1998; King et al., 2000; Gélineau et al., 2012), while the second corresponds to the manipulation of these estimates taking into account the characteristics of the distribution of the variables. There are multiple forms of simulation, which is why it is important to note that this definition has as a warning that it is not a necessarily unifying definition (King et al., 2000). The approach that we will follow is based on empirical simulations, whose main objective is to explore the distribution properties of the parameters and lead this information to the use of probabilities (King et al., 2000; Gélineau et al., 2012). We must mention that the methodology to be followed does not mean a solution to the endogeneity problems that we face, however, it allows us to carry out a more complete analysis by giving us the possibility of comparing probabilities in two different scenarios. By scenario, we consider a situation in which we assign a specific set of values to the independent variables in the model to obtain a predicted probability.

The benefit of this exercise is that it allows us to measure the impact of a particular variable on the predicted probability. To perform this exercise, the simulation is repeated keeping the variables at their same values except for the variable of interest, which varies freely within a certain range, for example, an increase of one unit in the case of a continuous variable. Although the method is extremely simple, it can be useful for representing realistic scenarios of interest and reporting the effect of certain variables (King et al., 2000). Our interest in statistical simulation is that it methodologically represents a bridge to counterfactual analysis. The logic of the latter is closely related to experimental language, treatment versus control, however this does not have to be limited to experimental designs, and it is at this point that observational analysis can emulate counterfactual logic. It is important to note that the above does not suggest that statistical simulations act as a substitute for experiments, but rather, we highlight that they can be designed using a counterfactual language, thus approaching experimental designs (Gélineau et al., 2012; Kästner and Arnold, 2012). Survey-based research uses random samples of the population to report certain characteristics, such as the mean or variance, the estimated values of which will be more accurate as the number of observations, n, in the data set increases. The simulations follow a similar logic, with the difference that we are informed by probability distributions instead of populations. The information of a distribution is obtained by simulating from it random numbers that allow us to draw an approximation of a certain characteristic of the probability distribution. The approximations can be computed with a certain degree of precision by increasing the number of simulations (King et al., 2000; Gélineau et al., 2012). The proposed methodology is based on the simulation of estimated parameters to then obtain predicted values, expected values and first differences. It is important to mention that the value of said parameters is not accurate because the sample is finite. As a consequence of this, it is necessary to capture this uncertainty by simulating a plausible number of parameter sets from the random draw of the sample distribution. Although the simulated values may differ from the estimated β, they are consistent with the sample and the statistical model (King et al., 2000). To simulate the parameters, it is necessary to have the estimates and their respective variance and covariance matrix, so we consider ŷ as the vector of $\hat{\beta }$ and $\hat{\alpha }$, $ŷ=vec\left(\hat{\beta },\hat{\alpha }\right)$, and $\hat{V}\left(ŷ\right)$ as the variance and covariance matrix associated with the estimates. Using the central limit theorem, with a large enough sample and a limited variance it is possible to simulate randomly the parameters of a normal distribution with mean ŷ and variance $\hat{V}\left(ŷ\right)$, so that:

(6) $$\begin{array}{l} ỹ\sim N\left(ŷ,\hat{V}\left(ŷ\right)\right) \end{array}$$

The simulation of *y* is based on the following steps (King et al., 2000):

1. Estimate a model that maximizes the probability function and obtain ŷ y $\hat{V}\left(ŷ\right)$.
2. Simulate a vector value *y* from the multivariate normal distribution, $ỹ=vec\left(\tilde{\beta },\tilde{\alpha }\right)$, (5).
3. This last step is repeated *M* = 1,000 times to obtain 1,000 “drawings” of the parameters.

That said, and even more specific, our interest in the proposed method lies in the possibility of simulating a value of *y* conditional on a certain value chosen for the independent variables, denoted as vector *X*_*c*_. Likewise the simulated value δ corresponds to ${\tilde{\delta }}_{c}$, while the simulated predicted value of *y* is ỹ_*c*_, it is this last value that we will use as a simulated counterfactual predicted value. The process involves the following stages (King et al., 2000):

1. Using the algorithm described above, a value for the vector is simulated $ỹ=vec\left(\tilde{\beta },\tilde{\alpha }\right)$.
2. Identifying the predicted value to simulate, the value for the independent variables represented by the vector is fixed *X*_*c*_.
3. Taking the simulated effect of the coefficients of the upper portion of ỹ, is computed ${\tilde{\delta }}_{c}=g\left(X_{c},\tilde{\beta }\right)$, where the function *g* is the systematic component of the statistical model.
4. Finally, the result variable is simulated ỹ_*c*_ taking a random drawings of $f\left({\tilde{\delta }}_{c},\tilde{\alpha }\right)$, stochastic component of the statistical model.

#### 4.2.1. Simulation Stages

Before presenting the results obtained, it is important to point out how the theoretical foundations mentioned in the previous section were applied. The first strategy consists of estimating the predicted probability of forming friendship establishing the values of all the explanatory variables included in the model around its mean, with this it is possible to calculate the average probability of occurrence of a positive value in the dependent variable, that is to say, propensity to form friendship. This analysis allows us to answer the question about the probabilities of forming friendship for an average student at the university, defined by their socioeconomic characteristics. Regarding this, the theory indicates that when using average values, this strategy assumes that the independent variables follow a normal distribution. The second strategy consists of estimating again the predicted value of forming friendship but instead of using the mean values of the sample the exercise involves working through iterations using the actual values observed for each individual in the data set. The first iteration uses the values of individual *i*, the second the values of individual *ii*, the third the values of individual *iii*, and so on. Finally, as many iterations as individuals are completed in the dataset. Final estimates are obtained by taking the predicted mean probabilities of the *n* iterations performed. It is important to note that the particularity of this method is that it reflects the actual values observed, so it does not assume that the independent variables follow a normal distribution. As a third strategy, the differences in the predicted probabilities (first difference) are estimated establishing the explanatory variables in their mean. Given that the research focuses on the effect of staying at university on the probability of forming friendships, it is of interest to describe the marginal effect of the *time*(time) variable, so the strategy used allows the latter to vary by reducing it by one standard deviation below average, thus capturing the effect of shorter time spent at the university on friendship formation. This is where counterfactual logic is used when comparing two scenarios since the variables have been manipulated through simulation. Although the first differences report information regarding the magnitude of a certain variable, the results estimated using the strategy described above continue to be limited by the values at which the independent variables were established, in this case by the mean. In order to obtain more robust results, in this fourth and last exercise we will work with iterations, as described in the second strategy. The exercise consists of degrading the explanatory variable of interest, in this case time, by one unit from the actual value observed for each individual. This means that for the first observation the time variable is decreased by one standard deviation from the real value, which is done successively with the rest of the sample. This is again where we turn to counterfactual logic. Therefore, the first difference is estimated for each observation in as many iterations as observations contained in the data set. The individual effects are averaged, so are the upper and lower confidence interval. We maintain that this strategy allows the predicted probabilities to be estimated with respect to the real values and, therefore, to be more robust.

#### 4.2.2. Applications and Results

Given that we are interested in the effect of the time spent at the university on the probabilities of forming friendship networks, in this section we will focus our attention on years completed in the program by the students. As mentioned in the previous section, the *time* variable is statistically significant at a level of 0.001, while the associated coefficient is positive, which suggests that the longer the time spent at the university, the greater the propensity to form friendship ties, when all other variables remain constant. As described above, the first post-estimation strategy is to obtain the predicted probability of forming friendship when all other explanatory variables contained in the model are adjusted to their mean value. The results of this exercise are contained in Table 5 simulation section 1, being 0.0006 the average of the predicted probability of forming friendship. This result is interesting in itself, however it has an implicit warning regarding the dichotomous variables included in the model. When it comes to dichotomous variables, the mean, although statistically correct, is simply not realistic (King et al., 2000). Given that in this case variables such as different types of financing and different types of school are dichotomous, it is appropriate to resort to an approach that is more representative with respect to the data (King et al., 2000; Gélineau et al., 2012).

**Table 5:** Counterfactual simulations.

***Simulation 1***			
Predicted probability of forming friendship when independent variables fit the mean			
	Mean	Standard error	Conf. Interval 95%
Pr (y=1)	0.00059	0.00005	0.00050; 0.00067
*Simulation 2*			
Predicted probability of forming friendship since observed values are imposed sequentially			
	Mean	Standard error	Conf. Interval %
Pr (y=1)	0.00321	0.00675	0.00277; 0.00370
*Simulation 3*			
Marginal effect of the time variable, since all independent variables are adjusted to their mean and the time variable is downgraded one standard deviation from its mean			
	Mean	Standard error	Conf. Interval 95%
dPr (y=1)	-0.00011	0.00002	-0.00016; -0.00007
*Simulation 4*			
Marginal effect of the time variable, since all the independent variables are adjusted to their mean and the time variable is downgraded one standard deviation from its observed value			
	Mean	Standard error	Conf. Interval 95%
dPr (y=1)	-0.00005	0.00010	-0.00007; -0.00003

The second strategy implemented sequentially imposes the values of each observation and then averages the predicted probability. As reported in simulation 2, the results indicate that the estimated probability of forming friendships is 0.0032, with a confidence interval of 0.0028 to 0.0037.

It is interesting to note that our second strategy results in a predicted probability that differs from that calculated in the first strategy, so it is important to note that these types of post-estimation methods are sensitive to the values we use to obtain the estimated probabilities. The results of simulations 3 and 4 correspond to the difference in the predicted probabilities, first differences, applying the two approaches previously described. This strategy estimates the marginal effect of time by setting all explanatory variables to their mean value allowing the time variable to vary by downgrading it one standard deviation from the mean. The marginal effect, in this case, is simply the reported difference between the two predicted probabilities for the two counterfactual scenarios. As a result, we observe that the marginal effect of time is −0.0001, with a confidence interval that goes from −0.0002 to −0.0001, which suggests that 1 year less in university means a decrease in the predicted probability of forming friendship. Finally, the marginal effect of the time variable is again estimated, being degraded, this time, iteratively by one standard deviation for each observation. The results of simulation 4 show that the marginal effect of time on the propensity to form friendship −0.00005, with a confidence interval that ranges from −0.00007 to −0.00003. Again, the results are aligned to the positive effect of the time spent in the university.

## 5. Discussion

We have analyzed social networks and friendship formation in a Chilean university. The aim has been to test if being part of the same institution increases the probability of two students of different socioeconomic background become friends. One possible underlying mechanism is the sense of belongingness that being part of the same institutions may produce amongst students. For instance, it has been shown that the sense of belongingness increases cross-cultural interactions between domestic and international students in American universities and it enhances international students' average grade earned.

Literature has indicated that environmental and individual characteristics are crucial drivers of friendship and that there is a tendency of forming friendship with similar others. Consequently, is common to observe segregation or homophily in friendship networks. Our results indicate that being part of the same institution increases the probability of two students of different socio economic background become friends. This observed homophily may have significant effects on those less advantaged students as networks play an important role in aspects of social and economic life of individuals such as well-being, learning, information transmission and labor market outcomes at the beginning of professional careers. For instance, regarding the latter it has been shown that better connected individuals will have the opportunity of having access to new information sooner and at the same time they will be able to have high level of influence on the eventual spread of information. Additionally, there is evidence on the positive effects that social connections have on health and longevity and on the fact that lacking social connections qualifies as a risk factor for premature mortality (Holt-Lunstad, 2018). Increasing friendship between students of different socioeconomic profile is not good just for the less advantaged students but for the society as whole. Greater levels of interaction between individuals of different social class would have as a result greater levels of generalized trust, and trust, as it has been documented in several investigations, is a key factor behind countries development. In this sense, a society where there is a greater degree of social mobility, and the relevance of the socioeconomic origin of individuals would not represent an obstacle to accessing better opportunities, boosts trust and development.

Albeit we have shown that the longer students are in university the greater the probability of students of different socioeconomic background become friends, it is very important to implement policies ables to encourage interaction amongst students of different social classes, like sport or social clubs as there are investigations pointing out that cultural events, leaderships programs, and community service enhance belongingness, buffer racism and provide a secure base for cross-cultural relationships.

## 6. Conclusions

Undoubtedly, the data on networks represent valuable information to understand how the interaction of agents affects a series of social phenomena. Segregation as well levels of homophilia within a network, determine a person self-development, as sense of belonging and levels of integration allow people to have access to different options to improve their lives, and so their well-being. Belongingness is the driving force to integrate the different parts of a social network, in particular by considering that our analysis claims university to be the context where students own the chance of strengthening their social networks. Peers influence and belongingness absence may determine more diverse as well less segregated social networking. The level of connection of a network is decisive in the learning processes, information dissemination, access to employment opportunities, social mobility, among other phenomena, which is why the study of networks is crucial on the road to a society with minor segregation levels. This document shows our interest in opening a window in the analysis of networks at the educational level in Chile, this as a contribution to the development and implementation of future policies that reduce socioeconomic gaps that hinder access to more and better opportunities both in education as in employability. The data provides us with an overview of the network formed at the university. Given that there is no university-wide network registry in the country, it is important to note that we are aware of the limitations and problems of endogeneity that having a cross-sectional set of data represents. However, our analysis shows many of the characteristics exhibited on social networks from similar research. In particular, we can reference the level of grouping, the positive degree correlation, variance and asymmetry in the degree distribution, all variables whose result is aligned with the evidence present in the associated literature. More specifically, we assess the level of student segregation, using variables of socioeconomic characterization. Our findings suggest that for certain categories the segregation is rather moderate, this occurs in the categories in which the crossing of different characteristics is analyzed. On the other hand, when analyzing the crossing of equal categories, segregation increases substantially since the fraction of friends with equal socioeconomic characteristics differs significantly from the fraction that would be generated through a process of random assignment of friends. Regarding the factors that determine the probability of forming friendship, gender, year of entry (cohort) and academic ranking of the students are significant variables. Regarding socioeconomic variables, the results are also significant, showing that different students in this aspect are less likely to form friendships. On the other hand, the variable time of stay in the university, referring to the years that the student has been studying, increases this probability. Having exposed the above, it seems relevant to us that the administrative authorities implement measures that better integrate their students, taking advantage of the positive impact of time on the probability of forming friendship. Using counterfactual simulation as a methodology to achieve a broader analysis of the data in a counterfactual scenario, we found evidence in favor of the results obtained previously. In particular, we find interesting the impact of time on the predicted probability of forming friendship even when we condition this value to certain socioeconomic characteristics. Specifically, the variable is positive even when the individuals come from different socioeconomic levels. It is important to highlight that the procedures used in the simulations have certain limitations that derive from the specification of the estimated model and the values assigned to the independent variables, so it would be ambitious to think that we can completely avoid endogeneity problems. That said, we leave the challenge for future research, in which it is possible to develop models that allow the implementation of more complex mechanisms of interaction between agents, incorporating in them a series of factors that are impossible to avoid when understanding network formation.

## Data Availability Statement

The raw data supporting the conclusions of this article will be made available by the authors, without undue reservation.

## Ethics Statement

The studies involving human participants were reviewed and approved by The University Diego Portales, Chile. The ethics committee waived the requirement of written informed consent for participation.

## Author Contributions

PL: data collection, questionnaire design, statistical analysis, counterfactual simulations, results interpretation, and writing. MV: questions and hypotheses formulation, methodology design, results' interpretation, and writing. CC: statistical analysis and writing. All authors contributed to the article and approved the submitted version.

## Conflict of Interest

The authors declare that the research was conducted in the absence of any commercial or financial relationships that could be construed as a potential conflict of interest.

## Footnotes

^1^In Chile, the results of research on social mobility indicate a relatively mobile and permeable middle class structure with a tendency to polarize, as social distances continue to increase despite economic growth. Specifically, the results suggest high mobility for the middle deciles and low mobility for the richest deciles in the distribution (Contreras et al., 2004). That said, the intervention of policies that manage to increase dynamism across the socioeconomic scale must consider all those situations or means in which the integration of the different structures of the social network is achieved, especially those that are more segregated.

^2^Chilean Ministry of Education.

^3^“The University Admission System: Commitment and Contribution of the Council of Rectors of Chilean Universities to higher education 2020.”

^4^For the analysis of these results see Table 4.1, where the decomposition of each category is presented.

^5^The authors developed a computer program called Clarify; software for the interpretation and presentation of statistical results, designed to implement in stata the methods that we use in this document. Available at http://GKing.Harvard.Edu.

## References

[B1] Adler P. Kwon S. (2002). Social capital: prospects for a new concept. Acad. Manage. Rev. 27, 17–40. 10.5465/amr.2002.5922314

[B2] Allende C. Valenzuela J. P. y Diaz R. (2018). School segregation in Chile, in Global Encyclopedia of Public Administration, Public Policy, and Governance, ed Farazmand E. A. (Springer International Publishing). 10.1007/978-3-319-31816-5

[B3] Baumeister R. F. Leary M. R. (1995). The need to belong: Desire for interpersonal attachments as a fundamental human motivation. Psychol. Bull. 117, 497–529. 10.1037/0033-2909.117.3.497 7777651

[B4] Blau P. (1977). Inequality and Heterogeneity. New York, NY: Free Press.

[B5] Boix C. Posner D. (1996). Making Social Capital Work: A Review of Robert Putnam's Making Democracy Work: Civic Traditions in Modern Italy. Harvard University Centre for International Affairs Working Paper Series, 96.

[B6] Brechwald W. Prinstein M. (2011). Beyond homophily: a decade of advances in understanding peer influence processes. J. Res. Adolesc. 21, 166–179. 10.1111/j.1532-7795.2010.00721.x 23730122 PMC3666937

[B7] Calvó Armengol A. Jackson M. (2004). The effects of social networks on employment and inequality. Am. Econ. Rev. 94, 426–454. 10.1257/0002828041464542

[B8] Calvó-Armengol A. Patacchini E. Zenou Y. (2009). Peer effects and social networks in education. Rev. Econ. Stud. 76, 1239–1267. 10.1111/j.1467-937X.2009.00550.x

[B9] Chmielewski A. Savage C. (2014). Socioeconomic Egregation Between Schools in the U.S. and Latin America, 1970-2012. Lincoln Institute of Land Policy, Cambridge, MA.

[B10] Choudhury S. Blakemore S. Charman T. (2006). Social cognitive development during adolescence. Soc. Cogn. Affect Neurosci. 1, 165–174. 10.1093/scan/nsl024 18985103 PMC2555426

[B11] Conley T. Topa G. (2003). Socio-economic distance and spatial patterns in unemployment. J. Appl. Econometr. 17, 303–327. 10.1002/jae.670

[B12] Contreras D. Cooper R. Herman J. Neilson C. (2004). Dinámica de la Pobreza y Movilidad Social: Chile. Departamento de Economia, Universidad de Chile, 1996–2001.

[B13] Currarini S. Jackson M. Pin P. (2009). An economic model of friendship: homophily, minorities, and segregation. Econometrica 77, 1003–1043. 10.3982/ECTA7528

[B14] Den Hartog D. De Hoog A. Keegan A. (2007). The interactive effects of belongingness and charisma on helping and compliance. J. Appl. Psychol. 92, 1131–1139. 10.1037/0021-9010.92.4.1131 17638470

[B15] DiPrete T. (2011). Segregation in Social Networks Based on Acquaintanceship and Trust. University of Chicago, Chicago, IL. 10.1086/659100 21648251

[B16] Durlauf S. (2002). On the empirics of social capital. Econ. J. 112, 459–479. 10.1111/1468-0297.00079

[B17] Elacqua G. (2012). The impact of school choice and public policy on segregation: evidence from Chile. Int. J. Educ. Dev. 32, 444–453. 10.1016/j.ijedudev.2011.08.003

[B18] Elacqua G. Contreras D. Salazar F. Santos H. (2011). The effectiveness of private school franchises in Chile's national voucher program. School Effect. School Improvement 22, 237–263. 10.1080/09243453.2011.589858

[B19] Espinoza Diaz O. González L. E. (2012). Pol?ticas de educación superior en Chile desde la perspectiva de la equidad. Soc. Econ. 68–94.

[B20] Flashman J. (2011). Academic achievement and its impact on friend dynamics. Sociol. Educ. 85, 61–80. 10.1177/0038040711417014 25705057 PMC4335644

[B21] Fletcher J. Ross S. (2012). Estimating the Effects of Friendship Networks on Health Behaviors of Adolescents. NBER Working Paper 11/13. York: Department of Economics, University of York. 10.3386/w18253

[B22] Fukuyama F. (1997). Social capital and the modern capitalist economy: creating a high-trust workplace. Stern Bus. Mag. 4, 1–16.

[B23] García Huidobro J. Bellei C. (2003). Desigualdad educativa en Chile, ed Hevia R. Santiago: Universidad Alberto Hurtado, Escuela de Educación„ 87–117.

[B24] Gélineau F. Bédard P. Ouimet M. (2012). Statistical simulation and counterfactual analysis in social sciences. Tutor. Quant. Methods Psychol. 8, 96–107. 10.20982/tqmp.08.2.p096

[B25] Glass C. R. Westmont C. (2014). Comparative effects of belongingness on the academic success and cross-cultural interactions of domestic and international students. Int. J. Intercult. Relat. 38, 106–119. 10.1016/j.ijintrel.2013.04.004

[B26] Golub B. Jackson M. (2012). How homophily affects the speed of contagion, best response and learning dynamics. Q. J. Econ. 127, 1287–1338. 10.1093/qje/qjs021

[B27] Goyal S. Van der Leij M. Moraga J. (2006). Economics: an emerging small world. J. Polit. Econ. 114, 403–412. 10.1086/500990

[B28] Granovetter M. (1995). The impact of social structure on economic outcomes. J. Econ. Perspect. 19, 33–50. 10.1257/0895330053147958

[B29] Holt-Lunstad J. (2018). Why social relationships are important for physical health: a systems approach to understanding and modifying risk and protection. Annu. Rev. Psychol. 69, 437–458. 10.1146/annurev-psych-122216-011902 29035688

[B30] Ioannides Y. Loury L. (2004). Job information networks, neighborhood effects, and inequality. J. Econ. Literat. 42, 1056–1093. 10.1257/0022051043004595

[B31] Jackson M. (2005). Group formation in economics: networks, clubs, and coalitions, in A Survey of Models of Network Formation: Stability and Efficiency, eds Demange G. Wooders M. (Cambridge, UK: Cambridge University Press), 11–57. 10.1017/CBO9780511614385.002

[B32] Jackson M. Rogers B. (2007). Meeting strangers and friends of friends: how random are social networks? Am. Econ. Rev. 97, 890–915. 10.1257/aer.97.3.890

[B33] Jackson M. Rogers B. Zenou Y. (2017). The economic consequences of social-network structure. J. Econ. Literat. 55, 49–95. 10.1257/jel.20150694

[B34] Jackson M. O. (2008). S ocial and Economic Networks. Princeton, NJ: Princeton University Press 10.1515/9781400833993

[B35] Kass R. Carlin B. Gelman A. Neal R. (1998). Markov chain monte carlo in practice: a roundtable discussion. Am. Statist. 52, 93–100. 10.1080/00031305.1998.10480547

[B36] Kästner J. Arnold E. (2012). When can a Computer Simulation act as Substitute for an Experiment? A Case-Study From Chemistry. Preprint Series, Stuttgart: Stuttgart Research Centre for Simulation Technology (SRC SimTech); University of Stuttgart.

[B37] King G. Tomz M. Wittenberg J. (2000). Making the most of statistical analyses: improving interpretation and presentation. Am. J. Polit. Sci. 44, 341–355. 10.2307/2669316

[B38] Lambiri D. Vargas M. (2011). Residential Segregation and Public Housing Policy, The Case of Chile. Documento de Trabajo de la Facultad de Economía y Negocios de la Universidad Diego Portales.

[B39] Lazarsfeld P. F. Merton R. K. (1954). Friendship as a social process: a substantive and methodological analysis, in Freedom and Control in Modern Society, ed Berger M. (New York, NY: Van Nostrand), 18–66.

[B40] Lee C. Scherngell T. Barber M. J. (2011). Investigating an online social network using spatial interaction models. Social Netw. 33, 129–133. 10.1016/j.socnet.2010.11.002

[B41] Mahar A. Cobigo V. Stuart H. (2013). Conceptualizing belonging. Disabil. Rehabitil. 35, 1026–1032. 10.3109/09638288.2012.717584 23020179

[B42] Marmaros D. Sacerdote B. (2006). How do friendships form? Q. J. Econ. 121, 79–111. 10.1162/qjec.2006.121.1.79

[B43] Maslow A. (1970). Motivation and Personality. New York, NY: Harper and Row.

[B44] Mayer A. Puller F. (2008). The old boy (and girl) network: social network formation on university campuses. J. Public Econ. 92, 329–347. 10.1016/j.jpubeco.2007.09.001

[B45] McPherson M. Smith Lovin L. Cook J. M. (2001). Birds of a feather: homophily in social networks. Annu. Rev. Sociol. 27, 415–444. 10.1146/annurev.soc.27.1.415

[B46] Mele A. (2017). A structural model of dense network formation. Econometrica 85, 825–850. 10.3982/ECTA10400

[B47] Mizala A. Torche F. (2012). Bringing the schools back in: the stratification of educational achievement in the Chilean voucher system. Int. J. Educ. Dev. 32, 132–144. 10.1016/j.ijedudev.2010.09.004

[B48] Mollica K. Gray B. Trevino L. (2003). Racial homophily and its persistence in newcomers' social networks. Organ. Sci. 14, 123–136. 10.1287/orsc.14.2.123.14994

[B49] Montgomery J. (1991). Social networks and labor-market outcomes: toward an economic analysis. Am. Econ. Rev. 81, 1408–1418.

[B50] Moody J. (2001). Race, school integration, and friendship segregation in America. Am. J. Sociol. 107, 679–716. 10.1086/338954

[B51] Newman M. (2003). The structure and function of complex networks. SIAM Rev. 45, 167–256. 10.1137/S003614450342480

[B52] Putman R. (1995). Bowling alone: America's declining social capital. J. Democracy 6, 65–78. 10.1353/jod.1995.0002

[B53] Royuela V. Vargas M. (2006). Segregación Residencial: Una revisión de la literatura. Universidad Diego Portales.

[B54] Rozer J. Hofstra B. Brashears M. Volker B. (2020). Does unemployment lead to isolation? The consequences of unemployment for social networks. Soc. Netw. 63, 100–111. 10.1016/j.socnet.2020.06.002

[B55] Saporito S. (2003). Private choices, public consequences: magnet school choice and segregation by race and poverty. Soc. Probl. 50, 181–203. 10.1525/sp.2003.50.2.181

[B56] Schneider M. Elacqua G. Buckley J. (2006). School choice in Chile: is it class or the classroom? J. Policy Anal. Manage. 25, 577–601. 10.1002/pam.20192

[B57] Smirnov I. Thurner S. (2016). Formation of homophily in academic performance: students change their friends rather than performance. PLos ONE 12:e0183473. 10.1371/journal.pone.0183473 28854202 PMC5576666

[B58] Trevino E. Valenzuela J. P. Villalobos C. (2014). Segregaciónn Académica y Socioeconómica al interior de la escuela: *M*agnitud, Evolución y Principales Factores Explicativos. FONIDE F711296 Ministerio de Educación.

[B59] Valenzuela J. Bellei C. De los Ríos D. (2008). Evolución de la Segregación Socioeconómica de los Estudiantes Chilenos y su Relación con el Financiamiento Compartido. Informe Final Proyecto Fonide.

[B60] Von Scheve C. Esche F. Schupp J. (2017). The emotional timeline of unemployment: anticipation, reaction, and adaptation. J. Happiness Stud. 18, 1231–1245. 10.1007/s10902-016-9773-6

[B61] Weber H. Schwenzer M. Hillmert S. (2020). Homophily in the formation and development of learning networks among university students. Netw. Sci. 8, 469–491. 10.1017/nws.2020.10

[B62] Xu L. Weinberg B. (2014). Unrequited friendship? How reciprocity mediates adolescent peer effects. Region. Sci. Urban Econ. 48, 144–153. 10.1016/j.regsciurbeco.2014.06.001

[B63] Yuan Y. Alabdulkareem A. Pentland A. (2018). An interpretable approach for social network formation among heterogeneous agents. Nat. Commun. 9:4704. 10.1038/s41467-018-07089-x 30410019 PMC6224571

[B64] Yuval-Davis N. (2006). Belonging and the politics of belonging. Patterns Prejudice 40, 197–214. 10.1080/00313220600769331

